# Adjunct Therapies to Standard Care for IBS and IBD Patients: Digestive Symptoms Improvement and Quality of Life Optimization

**DOI:** 10.3390/nu16223927

**Published:** 2024-11-18

**Authors:** Veronique Traynard

**Affiliations:** RNI-Product-Life Group, RNI Conseil, 17 Rue des 2 Haies, 49100 Angers, France; vtraynard@productlife-group.com

**Keywords:** inflammatory bowel disease, irritable bowel syndrome, medical nutrition therapies, probiotics, fibers, Mediterranean diet, physical activity

## Abstract

**Background:** The prevalence of both inflammatory bowel diseases (IBD) and Irritable Bowel Syndrome (IBS) is increasing, with persistent digestive symptoms, an altered quality of life, and higher rates of anxiety, chronic fatigue, and sleep trouble than the general population. **Methods:** This scoping review will analyze the latest clinical practice recommendations and clinical studies on non-pharmaceutical interventions such as diet adaptations, physical activity, cognitive behavioral therapies, and medical nutrition therapies such as probiotics, soluble fibers, chitin-glucan, and micronutrients for digestive symptoms relief, quality of life improvement and nutritional deficiencies correction in IBS and IBD patients. The objective is to help healthcare practitioners and dietitians to build personalized care program for IBD and IBS patients. **Results:** Mediterranean diet, physical activity, cognitive behavioral therapies and medical nutrition therapies such as selected probiotics, soluble fibers, chitin glucan, peppermint oil and micronutrients are effective as adjunct therapies. **Conclusions:** These adjunct therapies may help to reduce persistent digestive symptoms, correct nutritional deficiencies and improve quality of life of IBS and IBD patients.

## 1. Persistent Digestive Symptoms and Altered Quality of Life in IBS and IBD Patients

Inflammatory bowel diseases (IBD) includes three main pathologies: ulcerative colitis (UC), Crohn’s disease (CD), and unclassified IBD (IBD-U). The number of new IBD cases is constantly increasing. In 1990, there were 3.7 million of patients suffering from IBD globally, whereas in 2017, they reached 6.8 million [1]. On the one hand, about 50% of patients suffering from IBD display mild evolution in their disease, with a low prevalence of relapses and complications. On the other hand, the other 50% present a severe form with regular relapses, frequent hospitalizations, and the altered quality of life associated with intestinal complications, and resort to surgery [2,3].

There is an absence of correlation between endoscopic remission and clinical remission in IBD patients. Furthermore, even with an improvement or even a normalization of inflammatory markers (fecal calprotectin, CRP), a significant fraction of patients report an impaired quality of life due to persistent daily functional digestive disorders common to Irritable Bowel Syndrome (IBS) such as constipation, urgency/incontinence, rectal pain, and fatigue. Indeed, 40–60% of CD patients are in remission and 46% of UC patients display IBS-like symptoms [4,5], including abdominal pain and cramping, diarrhea or constipation, bloating, and flatulence. These persistent symptoms are correlated with an altered quality of life [4,5]:

- Anxiety is twice as high in people suffering from CD or UC than the general healthy population [6,7].
- A total of 50% display chronic fatigue when in remission and 72% when in the active phase of the disease [8,9,10,11].
- A total of 40–60% display depressive symptoms [12].
- A total of 62% reported chronic sleep troubles [13].

A total of 38.5% of patients with CD and 43.5% of patients with UC, i.e., less than a half of the patients, believe that their disease is well controlled by treatment. The main reasons for this statement is the absence of partial response to treatment, a loss of response to treatment over time, side effects, and the recurrence of gastrointestinal symptoms. In this context, there is a need for personalized care programs with adjunct therapies in addition to drug treatment, to focus on the relief of digestive symptoms and quality of life improvement. Moreover, the level of information that patients have on their disease is unequal. Follow-ups with the dietitian or other healthcare practitioners vary widely.

## 2. Actual Standard Drug Medication for IBD and IBS

The first line of treatment for IBS is osmotic laxatives for constipation, loperamide for diarrhea, and antispasmodics for abdominal pain. The second line of treatment consists of secretagogues (linaclotide, luiprostone, and tenapanor) for IBS-C, eluxadoline for IBS-D, and tricyclic antidepressants.

In cases of UC, 5-aminosalicylates (5-ASA) are most often prescribed in early forms and low to medium severity forms of the disease as an induction therapy. Not all patients respond to 5-ASA treatments. Approximately 20 to 25% of patients taking 5-ASA temporarily stop their use due to undesirable effects including abdominal pain, nausea, flatulence, headache, diarrhea, cold, and the exacerbation of the UC [14,15].

In CD, glucorticosteroids are favored to reduce the action of NFkB and the production of pro-inflammatory agents such as cytokines. But their uses are limited due to their medium and long-term undesirable effects, such as body weight gain, increased blood pressure, endocrine and bone disorders, hyperglycemia/diabetes, cataracts, glaucoma, and mood changes. They can also be used in UC in the event of the failure of 5-ASA.

Immunomodulatory treatments such as anti-TNFα, which specifically block inflammatory factors involved in the disease, are generally prescribed as first-line therapy for CD. Examples of anti-TNFα include infliximab, adalimumab, and golimumab. Ustekinumab may be also an option. It is a bivalent monoclonal antibody, designed to bind to the p-40 subunit of interleukin-12 (IL-12) and interleukin-23 (IL-23). These molecular messengers are involved in intestinal inflammation and other processes that cause intestinal damage. By blocking them, ustekinumab reduces the activity of the immune system and the symptoms of CD. Remission is achieved by 50% at 44 weeks compared to the placebo group (35%). However, 2/3 of the patients do not achieve clinical remission and the need to proceed to surgery remains frequent [16]. Approximately 40% to 55% of patients are considered to be non-responders to anti-TNFa treatment or will have a loss of response [17,18].

In CD and UC, another option is levedolizumab. This medication blocks the trafficking of leukocytes in the intestine by blocking alpha-4 beta-7 integrin. A meta-analysis of 10 cohorts, including patients with CD and UC, demonstrated a level of loss of response to treatment of 47.9 per 100 person-years of follow-up for CD and 39.8 per 100 people-years of follow-up for the UC, suggesting a high rate of loss of response under these treatments [19]. Thiopurines (azathiopurines and 6-meraptopurine) are immunomodulatory treatments for moderate to serious forms of CD or UC. Methotrexate may also be used for CD. Despite their efficacy, the challenges of these treatments are the potential side effects, and an absence or loss of response.

In the context of loss of response to actual treatments, their associated side effects, and persistent IBS-like digestive symptoms, as well as the high prevalence of anxiety, sleep disorders, and an altered quality of life for those suffering from IBS, UC, and CD, there is a growing need for adjunct therapies for these patients.

## 3. Summary of Scientific Evidence on Diet Efficacy for Digestive Symptoms Management in IBD Patients

Several diets have been studied for IBS and IBD such as high-fiber diets, vegetarian diets, gluten-free diets, low FODMAP diets, specific carbohydrate diets, and Mediterranean diets. The main clinical findings and strength of evidence are summarized in Table 1.

### 3.1. High Fiber Diet

A prospective study demonstrated that in patients suffering from CD, the consumption of a high-fiber diet reaching 24.3 g/d is associated with a 40% lower risk of developing CD [20,21]. A meta-analysis including observational studies demonstrated that a diet rich in fiber may reduce the risk of developing IBD. Moreover, each 10 g/d increase in fiber is linked to a 13% decrease of CD risk [22]. A second meta-analysis including eleven studies on 2389 patients suffering from CD confirmed that consuming a high-fiber diet, with or without standard care, enhanced CD remission rates [23]. Fiber exerts an anti-inflammatory action through butyrate, has a beneficial action on the microbiome, and reduces intestinal permeability.

### 3.2. Specific Carbohydrate Diet

The Specific Carbohydrate Diet (SCD) allows the consumption of monosaccharides, and excludes disaccharides and most polysaccharides, in order to minimize digestive processes. It also eliminates complex polysaccharides. Fruits, most vegetables, meat, seafood, and hard cheeses are preferred. A clinical trial has shown that the SCD is not inferior to the Mediterranean diet for triggering remission in adults suffering from CD [24]. Specific nutritional deficiencies may be present in IBD patients because some foods are restricted or avoided, particularly dairy foods rich in vitamin D and calcium. The low consumption of grains, fruits and vegetables may trigger vitamin B1, vitamin B6, vitamin C, vitamin A and folate insufficiencies. A pilot study carried out by Cohen et al. in demonstrated that 33% of the study volunteers lost weight with the SCD [25]. Even if the SCD diet is associated to potential digestive symptoms improvement, additional studies, especially in adults, are needed to draw conclusions on the long-term benefits of the SCD. Also, long-term compliance to a restrictive diet and implications on nutritional status need to be addressed.

### 3.3. Low-FODMAP Diet

The low-FODMAP diet (low in Fermentable Oligosaccharides, Disaccharides, Monosaccharides and Polyols) avoids specific type of foods that are not well digested and are highly fermented, as the origin of flatulence and gastrointestinal symptoms. It is structured into three different steps: elimination, reintroduction, and maintenance [26]. The low-FODMAP diet triggers a significant reduction in digestive symptoms such as diarrhea, bloating, and abdominal pain in mild to moderate forms of IBD with stabilized treatment or patients in remission. Drugs that might alter, reduce, or modulate symptoms (such as laxatives), and antibiotic or probiotic consumption in the last 8 weeks before inclusion were also considered as an non-inclusion criteria [27,28]. Prince et al. showed that the low-FODMAP diet triggers a significant decrease in abdominal pain, bloating, and diarrhea in 72% of patients suffering from CD and 78% of patients with UC in remission [29]. Another study included 200 patients without active inflammation of IBD with IBS symptoms to evaluate the efficacy of the low-FODMAP diet. The use of probiotics, antidiarrheal medication, H2-receptor blockers, or proton-pump inhibitors was forbidden and a change of medication 1 month before the inclusion and during the study was considered as non-inclusion criteria. A low-FODMAP diet was consumed for 6 weeks. The low-FODMAP diet reduced the digestive symptoms such as intestinal gas and diarrhea without reducing the frequency of constipation [30]. Moreover, Bodini et al. carried out a randomized controlled trial where 55 adult patients with IBD and stabilized treatment consumed for 6 weeks a low-FODMAP diet or a standard diet. Lower calprotectin levels, decreased disease activity, and an improved quality of life were observed in the treatment group compared to the control group [31]. An initial meta-analysis including six randomized controlled trials (RCTs) and sixteen non-randomized interventions assessed the efficacy of the low-FODMAP diet on IBS-like symptoms in quiescent IBD patients. A significant reduction in IBS Symptom Severity Scale (IBS-SSS) scores for the group on a low-FODMAP diet was found in both the RCTs and non-randomized trials. Moreover, the IBS quality of life score was significantly improved in the low FODMAP group in both the RCTs and non-randomized trials. A low-FODMAP diet significantly decreased the severity of abdominal pain and global symptoms in the RCTs [32]. A second meta-analysis [33] confirmed that a low-FODMAP diet reduced functional gastrointestinal symptoms, improved Short inflammatory bowel disease questionnaire scores, and lowered the Harvey-Bradshaw index of CD.

However, foods high in FODMAP may trigger nutrient insufficiencies or deficiencies, mainly iron, calcium, zinc, folic acid, and vitamin D, as well as complex carbohydrates or antioxidants. Moreover, the consumption of prebiotics, fiber, fructo-oligosaccharides, and galacto-oligosaccharides is decreased in the low-FODMAP diet. This can alter the gut microbiota, the synthesis of short-chain fatty acids, and reduce their protective role on colon cells. Dysbiosis characterized by a lower concentration of *Bifidobacteria* spp., *Akkermansia muciniphila*, and *Clostridium cluster XIVa* has been observed in the low-FODMAP diet. Low-FODMAP diets may also be linked to insufficient energy intakes, enhancing the risk of malnutrition, the prevalence of which is frequent in patients suffering from IBD [34].

### 3.4. Vegetarian Diet and Gluten-Free Diet

A study recruited 1254 patients from the Swiss IBD Cohort Study with prospective data collection between 2006 and 2015. Eating habits were evaluated through a self-report questionnaire. Vegetarian diets and gluten-free diets in IBD patients are linked with poorer psychological well-being and dysbiosis, but no clinical benefits for the evolution of the pathology. Gluten-free diet patients had significantly higher levels of depressive symptoms or anxiety [35]. Although this diet may be associated with CRP decrease, additional studies are required to evaluate the benefits of vegetarian diets for IBD patients.

A cross-sectional study of 1647 patients with IBD recruited in a longitudinal internet-based cohort using a gluten-free diet questionnaire assessed the efficacy of such a diet for IBD patients [36]. A total of 65.6% of all patients on a gluten-free diet mentioned an improvement in their digestive symptoms and 38.3% reported fewer or less severe IBD flare ups. In the group on a gluten-free diet, high adherence was linked with significant fatigue reduction (*p* < 0.03). Further randomized controlled trials are required to further assess gluten-free diet efficacy for IBD patients.

### 3.5. Mediterranean Diet

The Mediterranean diet is characterized by the high consumption of olive oil, raw vegetables and fruits, nuts, dairy products, and fish, with a low consumption of red meat and processed foods, rich in saturated fat, and simple sugars. Because of its high content of antioxidants, the Mediterranean diet exerts anti-inflammatory effects. The Mediterranean diet may also be associated with a beneficial action on the microbiome, with more anti-inflammatory bacteria and less proinflammatory bacteria. A study carried out by Marlow et al. has demonstrated that the Mediterranean diet leads to a significant decrease in proinflammatory markers and the correction of dysbiosis in patients with IBD. Chicco et al. performed a prospective interventional trial on adults with IBD and stabilized treatment on the Mediterranean diet for 6 months. Reduced BMI and waist circumference, as well as decreased hepatic steatosis were observed. The anthropometric measures were correlated with improvements in disease activity, reduced CRP, and fecal calprotectin in patients with CD and UC [37]. A decrease in fecal calprotectin levels was also observed in adults with UC after pouch surgery who adhered to the Mediterranean diet. It is also associated with reduced disease activity and decreased inflammatory markers. A significantly lower number of patients in the active disease phase was also observed for patients on the Mediterranean diet. A prospective observational study included 153 patients with UC after pouch surgery on the Mediterranean diet. Adherence to the Mediterranean diet was associated with reduced fecal calprotectin levels [38]. In a retrospective study on 86 outpatients with CD, 80 CD patients who followed a Mediterranean diet displayed an improved quality of life [39] and lower disease activity [40]. These findings were confirmed in other prospective studies where a 6-month adherence to the Mediterranean diet was linked to a reduction in body weight and waist circumference. The proportion of CD patients in the active phase was significantly lower after 6 months compared to the baseline. Inflammatory markers were also significantly lower [37]. In another study, Mediterranean diet adherence score was significantly correlated with the IBD Questionnaire and inversely correlated with disease activity in 86 patients with CD (41 in relapse and 45 in remission) [39].

Dysbiosis is present in IBD and the Mediterranean diet modulates intestinal microbiota, enhancing beneficial bacteria, which increase gut barrier function and decrease inflammation. A Mediterranean-like dietary pattern decreases intestinal inflammation. Higher adherence to a Mediterranean diet is linked with a reduced risk of later-onset CD [2]. Polyphenols increases the growth of beneficial bacteria such as bifidobacteria and lactobacillus. Gut microbiota changes can reduce intestinal inflammation. The Mediterranean diet meets the clinical practice recommendation for IBD, including the decreased consumption of red meat and myristic acid while favoring the intake of omega-3 fatty acids. In conclusion, adherence to a Mediterranean diet triggers clinical benefits in the active phase of CD and is associated with lower levels of inflammatory markers in UC. It also improved quality of life and is associated with decreased mortality rates in IBD patients. Indeed, the Mediterranean diet promotes gut microbiota diversity and increases the growth of beneficial bacteria, exerts anti-inflammatory action through polyphenols and unsaturated fats, and reduces oxidative stress [41]. A prospective cohort study including 83,147 volunteers between 45–79 years recruited in the Cohort of Swedish Men and Swedish Mammography Cohort assessed the correlation between the Mediterranean diet and the risk of later-beginning of UC or CD. A higher modified Mediterranean diet adherence score, assessed by food frequency questionnaire, was associated with a reduced risk of CD [42].

A new food pyramid has been proposed by Rondanelli et al., characterized by five portions of fruits and cooked vegetables, three portions of grains, extra virgin oil, and yoghurt every day, four portions of fish every week, three portions every week of eggs and white meat, two portions every week of seasoned cheese, and one portion of red meat per week. A supplementation with omega-3, vitamin D, and calcium is advised in the food pyramid for IBD patients [43]. This is in line with the characteristics of the Mediterranean diet and previous studies reported a higher risk of flare ups in UC associated with the consumption of red meat, as well as a lower endoscopic activity in UC associated with a higher intake of vegetables and fruits [44].

## 4. Personalized Program with Physical Activity and Cognitive Behavioral Therapies for IBS and IBD Patients

### 4.1. Physical Activity

Physical activity is associated with several clinical benefits in IBS and IBD patients [45]. However, IBD patients tend to be less active than healthy people. The clinical benefits associated with physical activity reported in these patients are mainly: mood enhancement, fatigue reduction, and improved quality of sleep and perceived stress. These clinical benefits are particularly relevant as IBD patients are at risk of depressive symptoms, chronic fatigue, and sleep troubles. Moreover, physical activity has been associated with improved quality of life, reduced risk of active-phase flare ups, and reduced inflammatory markers in these patients. Davis et al. reviewed the clinical benefits of physical activity in IBD patients across 28 studies with a total of 8168 adults [46,47]. The review confirmed an improvement in QOL in seven studies, reduced mental and physical fatigue scores in five studies, and improved sleep quality, digestive symptoms, and cardiorespiratory performance. The review also identified physical activity as an inexpensive way to reduce IBD-associated sarcopenia and obesity-related metabolic alterations. Moreover, participation in physical activity decreased the risk of developing of IBD and the risk of flare up in five studies. The risk of CD onset is decreased by 44% in women who practiced 9 h of walking weekly in a longitudinal cohort across 2 years [48]. In a longitudinal study, higher levels of physical activity were significantly linked with a decreased risk of active CD in adults with CD in remission (*p* = 0.02) [49]. Moreover, 41.3% of adults with IBD (*n* = 158) in a cross-sectional survey reported that physical activity contributes to a decrease in their relapse rates [50].

Eckert et al. reviewed the clinical benefits of physical activity in IBD patients. Thirteen eligible articles were selected. Five studies assessed aerobic exercise, three trials evaluated resistance exercise, three studies assessed mind–body therapies, and two trials evaluated yoga. The patients had a significant increase in fitness, bone mineral density, and quality of life, and a decrease in IBD-related stress and anxiety [51].

A meta-analysis confirmed the beneficial effects of physical activity in IBD patients. Fifteen studies were analyzed, among which nine RCTs were selected, including 637 participants (64% female) [52]. Pooled data from six RCTs demonstrated that physical activity improved disease activity compared to control groups. The largest effect on disease activity was seen with a low intensity walking program. Benefits were significant in terms of tiredness, muscle strength, body composition, cardiorespiratory performance, bone mineral density, and psychological well-being. A meta-analysis of 28 studies, including four RCTs and 8168 adults, assessed the clinical benefits of physical activity for IBD patients [47]. Results from the majority of the trials reviewed confirmed the benefits of moderate-intensity physical activity for adults with IBD. Studies have demonstrated the following clinical benefits for IBD patients: improvement in quality of life, mental health, sleep quality, gut symptoms, tiredness, and cardiorespiratory capacity. Physical activity also decreased the risk of developing IBD and the risk of future active disease and relapse. The main barriers to engagement in exercise are tiredness, joint discomfort, abdominal pain, intestinal emergency, active illness, and depressive symptoms.

### 4.2. Cognitive and Behavioral Therapies

Complementary to physical activity, cognitive and behavioral therapies demonstrated significant benefits for IBD patients, such as a better ability to cope with illness [53], reduced anxiety, stress, and depression scores [54,55], and improved quality of life [56,57].

A review of 31 studies (32 articles) were selected and included 2397 patients with active IBD or in remission [58]. Eleven studies demonstrated a significant beneficial effect. Treatment type varied in the selected trials and consisted of stress management programs, cognitive behavioral therapy, hypnosis, psychodynamic therapy, solution-focused therapies, acceptance and commitment therapy, and mindfulness. The four studies carried out on patients with active disease demonstrated the beneficial effects of cognitive and behavioral therapies. Such interventions can improve quality of life for patients with IBD.

A recent randomized, controlled study was conducted on 60 adolescents and young adults with IBD in remission. Patients were randomly placed into either psychodynamic psychotherapy in addition to standard medical therapy or standard medical care alone for 8 weeks. Intention-to-treat analysis demonstrated significant improvement in maintaining IBD remission rates in the group undergoing short-term psychodynamic psychotherapy compared with the control group. The proportion of patients still in steroid-free remission after 52 weeks was significantly higher in patients in the group undergoing short-term psychodynamic psychotherapy (93.1%) compared with control group (64.3%; *p* = 0.01). There were also a significant reduction in depression symptoms in the group undergoing psychodynamic psychotherapy compared to the standard medical therapy group [59].

Cognitive behavioral therapies may be useful to develop adaptive coping skills, reduce IBD-related stress, and improve quality of life [60,61]. Mindfulness-based therapies [57], hypnosis [62], and stress management [63,64] also have encouraging clinical benefits for IBD patients in terms of reducing abdominal symptoms, and anxiety and depression scores. It may also prolong remission in patients with IBD [61].

## 5. Medical Nutrition Therapies Improve Digestive Symptoms and Correct Nutritional Deficiencies in IBS and IBD Patients

### 5.1. Chitin Glucan

The consumption of chitin glucan, an insoluble fiber of fungal origin, administered at the human equivalent doses of 1.5 to 3 g/d in a model of chronic colon hypersensitivity in Sprague Dawley rats for 12 weeks is associated with decreased visceral pain perception. A reduction in inflammation intensity by 50% was also observed, associated with a full regeneration of the colonic mucosa in the group treated with Dextran sulfate sodium-induced colitis. At a daily dose 3.0 g/d of chitin-glucan (human equivalent dose), the analgesic properties surpassed the effect of the spasmolytic drug phloroglucinol, occurring faster within 3 weeks and triggering a 50% inhibition of pain perception (*p* < 0.0001). The mechanism of action associated with these analgesic and anti-inflammatory effects of chitin glucan involved, partly, a significant induction of µ-opioid receptor, cannabinoid 2 receptor, and IL-10, and a significant inhibition of IL-1β and IL-8. Chitin glucan also significantly increased barrier-related genes such as claudin-2, mucin 5AC, and zonula occludens-2 [65]. In an open-label study, 120 patients with IBS consumed daily three sticks of a product containing 1.5 g of chitin glucan and 0.75 mg of simethicone for 4 weeks. At week 4, an improvement in abdominal pain was observed in 67% of the participants (score: 5.8 ± 2.4 vs. 2.9 ± 2.0, *p* < 0.0001). Moreover, bloating and abdominal distension were significantly reduced, and an improvement in the impact of global symptoms on daily life was also observed. Stool consistency improved in the majority of patients (90% for those with liquid stools and 57% for patients with hard stools, respectively) (Table 2) [66].

### 5.2. Selected Probiotics

Probiotics are live microorganisms that, when consumed in a significant amount, confer a health benefit to the host [67]. The Italian Ministry of Health has ruled in favor of the use of probiotics in the food industry over the past 12 years and has authorized the use of the word probiotic for foods in 2013 under specific conditions, such as a minimum number of viable cells (1 × 10^9^ CFU) consumed daily, a complete genetic characterization of the probiotic strain, and a demonstratable history of safe use in Italy. France also authorized the use of probiotics as a category name with an associated authorized health claim “participate to the balance of intestinal microbiota” under certain conditions: a minimal daily dose of 10^7^–10^9^ CFU, a proper characterization of the strain with an absence of antibioresistance, a safety guaranteed in the conditions of use, and a confirmed history of use in Europe. Several probiotics have been clinically tested in IBS and IBD adult populations for the relief of digestive symptoms. The use of the term “probiotics” is not harmonized across European Member States or the globe in the food supplements market.

A meta-analysis including 13 RCTs assessed the impact of probiotics on the reduction of digestive symptoms of IBS. The consumption of *B. coagulans* and *Bifidobacterium infantis* was associated with significant improvement in the global symptom scores in the treated group compared with the placebo group. *B. coagulans* ranked first in the relief of abdominal pain scores, while *S. cerevisiae* ranked second, *C. butyricum* ranked third, and *S. boulardii* ranked last. *B. coagulans* was effective in improving symptom relief rate, global symptoms, and reducing bloating, abdominal pain, and straining scores (Table 2). Moreover, *L. acidophilus* consumption was associated with a lower incidence of adverse events compared with those who were administered other treatment [68].

In an open-labeled, post-marketing study, the efficacy of *B. Longum* 35624 was assessed in 33 IBS adult patients from all IBS subtypes who were not consuming symptomatic drug medication for IBS. The supplementation of *B. longum* 35624 was linked to a significant decrease (43.4%) in the Total IBS Symptom Score (TISS) compared to baseline. The mean symptom severity degree relative to the passage of gas and bloating reduced significantly from “moderate” at baseline to “very mild to mild” after 8 weeks, while those for abdominal pain and diarrhea decreased significantly from “mild to moderate” to “very mild to mild.” More than 60% of the patients achieved clinically relevant decreases in the TISS (>30%) and in the IBS Symptom Severity Scale (IBS-SSS) score (>50 points) [69]. In an open-labeled observational study on 233 IBS patients, patients received one capsule of *B. longum* 35624 (10^9^ CFU) daily for 1 month. Antibiotic consumption or modification in the drug prescription that may trigger an effect on gut microbiota, intestinal motility, or gastrointestinal symptoms were not authorized. A significant reduction in IBS symptom severity was observed compared to baseline and 57% of patients were classified in lower severity grades or achieved remission. The quality of life of IBS patients was also enhanced by the treatment [70]. A real-world, open-labeled, single-arm trial carried out in Chile assessed the impact of *B. longum* 35624 administered at the daily dose of 1 × 10^9^ CFU for 12 weeks on gut symptoms in 64 children and adolescents (8–18 years) with IBS. The use of histamine type-2 receptor antagonists, proton-pump inhibitors, fermented foods, or probiotics within two weeks before the inclusion, or antibiotics within three months before inclusion was a criteria for non-inclusion [71]. Improvements in all IBS-SSS domains and composite scores were significant at weeks 6 and 12 (*p* < 0.0007 versus baseline), with 98.3% of IBS patients experiencing improvements in ≥3 domains. Clinically relevant improvement was observed in 96.6% of IBS patients. The distribution of IBS symptoms severity grades improved from moderate/severe at the beginning to mild or remission at the end of the study (*p* < 0.0001).

Another randomized, placebo-controlled double-blinded trial assessed the efficacy of two microorganisms on abdominal pain severity and digestive symptoms in IBS patients. A total of 330 patients with IBS aged 18 to 70 years were randomized (1:1:1) to consume *L. acidophilus* DDS-1 (1 × 10^10^ CFU/day), *B. animalis* subspp. *lactis* UABla-12 (1 × 10^10^ CFU/day), or a placebo for six weeks. The consumption of probiotic or fiber supplements or enriched foods, and IBS symptomatic medication were not authorized in the last month before inclusion. Laxative drugs or other medications affecting gastrointestinal motility were not authorized 2 weeks before inclusion. All were prohibited during the study. Significant improvement was observed in IBS-SSS scores for the *L. acidophilus* DDS-1 (−133.4 ± 95.19, *p* < 0.001) and *B. lactis* UABla-12 (−104.5 ± 96.08, *p* < 0.001) groups. Individual scores were improved for abdominal pain, bowel habits, and abdominal distension compared to the placebo group. Moreover, an improvement in stool consistency was significant in both probiotic groups after 6 weeks compared to the placebo group [72].

A randomized, single-blinded clinical study randomly assigned 100 IBS-D patients into two groups. The first group was administered a standard IBS treatment and the second group was treated with probiotics composed of *Lactobacillus acidophilus* and *Lactobacillus plantarum* in addition to the standard treatment. Both groups were supplemented for a period 12 weeks. The group receiving probiotics displayed higher reductions in IBS-SSS global scores. The individual item scores were also improved. The supplementation of both strains also improved disease severity and its associated symptoms if combined with the standard treatment [73].

A randomized, placebo-controlled clinical trial assessed the efficacy of a probiotic combination of *L. plantarum* CECT7484 and CECT7485 and *Pediococcus acidilactici* CECT7483 (3.10^9^ CFU/day), with or without the combination of the alverine (60 mg)/simethicone (300 mg), for the improvement of quality of life and the relief of digestive symptoms in patients with IBS aged 18–59 years. The response rate for IBS-Quality of Life was 50.0% for the probiotic group, 68.4% for patients who consumed probiotic combined with alverine/simethicone, and 16.7% in the placebo group after 6 weeks (*p* = 0.005). The group with probiotics and antispasmodics displayed the greatest improvement in quality of life (17 ± 13 points), followed by the probiotic group (15.12 ± 12.5 points) and the placebo group (8 ± 6.4 points). The response rate in abdominal pain, defined as a >30% reduction, was reported by 38.9% of the probiotic group, by 57.9% of the group who consumed probiotic combined with antispasmodic, and by 16.7% of the placebo group (*p* = 0.035). A response to treatment, defined for stool frequency as an average score < 5 on the Bristol Scale, was reported by 44.4% of patients who consumed the probiotic combination, by 57.9% of those who were administered the probiotic and antispasmodic combination, and by 16.7% in the placebo group (*p* = 0.032) [74].

*Bacillus coagulans* consistently improved digestive symptoms such as bloating, constipation, abdominal pain, rumbling, nausea, and vomiting in IBS patients in several randomized controlled trials for consumption between 8 to 12 weeks [75,76,77,78]. *Bacillus coagulans* supplementation in IBS with diarrhea also decreased the number of daily bowel movements after 8 weeks compared to a placebo group in a double-blinded randomized controlled trial [79].

A randomized, double-blinded, placebo-controlled clinical trial was carried out on 456 adults. After a run-in period, participants were randomized to receive probiotic *S. cerevisiae* I-3856 at the daily dose of 8 × 10^9^ CFU or a placebo for 8 weeks. They filled daily self-assessments of digestive symptoms. Participants consuming symptomatic medication modulating intestinal sensitivity or motility, such as opioids and narcotic analgesics, were not included in the study. However, laxatives, anti-bloating drugs, antispasmodics, antidepressants, anxiolytics, pain-killing drugs, and nonsteroidal anti-inflammatory medication were authorized only if consumed for more than 90 days and maintained at a stable daily dose during the whole study. A significantly greater proportion of abdominal pain responders reported an improvement in the probiotic group (45.1% vs. 33.9%, *p* = 0.017). After 8 weeks, global quality of life scores were significantly improved in the probiotic group compared to the placebo group [80]. This finding was confirmed in another RCT where 100 newly diagnosed subjects with IBS were classified into IBS-D, IBS-C, and IBS-M categories and randomly assigned to Saccharomyces cerevisiae CNCM I-3856 or placebo groups. Both treatments were administered in addition to standard therapy for 8 weeks. The average decrease in abdominal pain score in the probiotic group was significant when compared to the placebo group (*p* < 0.001). Significant improvement was also observed in the sub-categories. The stool consistency in IBS-D subgroup was significantly improved in probiotics group when compared to the placebo group at the end of the treatment (*p* < 0.001). Similar results were seen in the IBS-C and IBS-M subgroups [81].

In conclusion, selected probiotics such as *B. longum* 35624, *L acidophilus*, *L. plantarum*, *Bacillus coagulans*, *S. cerevisiae* and a combination of several strains are associated with statistically significant improvements in digestive symptoms such as abdominal pain and bloating, QOL improvement, reduction in IBS severity score, and the normalization of stool consistency and frequency. Some strains have specific mechanism of action such as reduction in visceral hypersensitivity, anti-inflammatory action, immune modulation, modulation of serotonin reuptake, and dysbiosis correction [82,83]. A striking change due to the increasing scientific evidence of the benefits of probiotics in IBS management is the progressive update of clinical practice recommendations: USA (Experts of Yale Workshop on Probiotics/USA/2011, 2015, American College of Gastroenterology 2014 and 2018), German (German Society for Digestive and Metabolic Diseases (DGVS and DGNM) in association with other German medical societies), French (SFNGE (2016, France), Japan Japanese Society of Gastroenterology 2021), British (British Dietetic Association 2012, 2016, and 2021), Korean (Korean Society of Neurogastroenterology and Motility (KSNM)/Korea/2018), Canadian (Canadian association of Gastroenterology 2019), Romanian (Romanian Society of Neurogastroenterology 2021), Belgian (Belgian clinical recommendations IBS), Indian (Indian consensus—IBS clinical practice recommendations 2023) recommendations favor probiotic use in clinical practice for IBS patients. The selection of the probiotic strain or combination needs to be carefully reviewed based on clinical evidence (daily dose, duration of use). The beneficial effect is strain-dependent so the clinical evidence cannot be extrapolated to other strains of the same species.

### 5.3. Soluble Fibers for Digestive Symptoms and IBS-like Symptoms in IBD

HMO (Human Milk oligosaccharides) supplementation displays promising clinical benefits in balancing the intestinal microbiota and improving IBS symptoms by stimulating the growth of beneficial bifidobacteria [84]. A multicenter, open-labeled trial included patients suffering from IBS from 17 US sites. Patients consumed daily 5-g of the HMOs 2′-fucosyllactose and lacto-N-neotetraose combination in a 4:1 blend for 12 weeks. IBS patients displayed significant improvement after 12 weeks in total percentage of bowel movements with abnormal stool consistency, overall IBS Symptom Severity Scale and health-related QOL compared to the placebo group. Similar improvements were observed for all IBS subtypes. Symptoms improved mainly during the first four weeks of treatment [85].

Galactooligosaccharides supplementation for IBS patients also demonstrated efficacy at daily doses of 3, 5, or 7 g/d after 12 weeks. Patients whose treatment was not stable in the past 3 months before inclusion or who were consuming probiotics or prebiotics 2 weeks before inclusion were excluded. The prebiotic significantly enhanced fecal bifidobacteria (3.5 g/d *p* < 0.005; 7 g/d *p* < 0.001). The placebo did not change the clinical parameters assessed, whereas the prebiotic at a daily dose of 3.5 g significantly improved stool consistency, reduced flatulence, and bloating. It also improved the composite scores of symptoms and subject global assessments. The galactooligosaccharides supplementation at a daily dose of 7 g per day significantly improved Subjective Global Assessment scores and anxiety scores [86].

A randomized controlled trial assessed the efficacy of sc-FOS and probiotics for IBS adult patients. Patients who were administered antibiotics during the study also were not included. Low doses of anti-depressants or spasmolytic drugs were authorized. A supplementation with a combination of sc-FOS and probiotics for 8 weeks triggered significant improvement in IBS-SSS and IBS global symptoms on the Global Improvement Scale (IBS-GIS). It also improved domain-specific scores relative to flatulence (*p* = 0.028) and bowel habits (*p* = 0.028) after 4 and 8 weeks. Patients who consumed synbiotics reported a significant improvement in the feeling of incomplete intestinal movements, abdominal pain, intestinal gas, and diarrheal stools compared to placebo group [87].

Another symbiotic combination, *B. longum* W11 and a soluble fiber Actilight^®^, a fructoligosaccharides from beetroot, has demonstrated efficacy for IBS. A total of 636 volunteers (250 men, 386 women) diagnosed with constipation-type IBS were recruited in 43 centers and received the symbiotic combination at a daily dose of 3 g for 36 days. Any drugs likely to alter gastrointestinal motility or antibiotics were withdrawn at the start of the study. The proportion of “no symptom” class increased significantly after the synbiotic treatment from 3% to 26.7% and from 8.4% to 44.1% for bloating and abdominal pain, respectively (*p* < 0.0001). In the more severe symptoms categories (moderate-severe), symptom frequency decreased significantly from 62.9% to 9.6% for bloating and from 38.8% to 4.1% for abdominal pain. Stool frequency improved significantly from 2.9 ± 1.6 times/week to 4.1 ± 1.6 times/week [88].

Belgian clinical practice recommendations (2022), the American Gastroenterological Association, and Indian clinical practice recommendations (2023) mentioned soluble fibers in the management of digestive symptoms for IBS patients. In Indian clinical practice recommendation, Ispaghula husk is specifically cited as a soluble fiber recommended for IBS-C patients. Psyllium husk supplementation enhances the abundance of intestinal microbiota producing short-chain fatty acid, suggesting the beneficial impact of using soluble fibers. They form gels in the intestine, increasing stool bulk and improving intestinal transit. Psyllium husk may also modulate the gut microbiota, improving its structure and function, and increasing the growth of beneficial bacteria (*Lactobacillus* spp. and *Bifidobacteria* spp.). Soluble fibers decrease abdominal pain and bloating, and improve transit. The positive effects of soluble dietary fiber have been demonstrated for both IBS-D and IBS-C [89].

### 5.4. Correction of Nutritional Deficiencies

IBD and IBS patients often display nutritional deficiencies, mainly in vitamin D, vitamin B6, B9, B12, B1, B12, iron, magnesium, and zinc [90]. A low-FODMAP diet or food avoidance may increase malnutrition or nutritional deficiency risks in adults. Medical nutrition therapies may correct these nutritional deficiencies. A supplementation of 50 µg/day of vitamin D is associated with nutritional status improvement for IBS patients and also an improvement in IBS-SSS. These findings are confirmed in two meta-analyses [91,92]. Vitamin D, B6, and zinc status are linked to IBS severity score. Magnesium salts may improve stool consistency and frequency. Zinc is involved in intestinal immune modulation and intestinal gut barrier integrity. Vitamin B6, B9, and B12 are also involved in intestinal immune modulation. Reduced serum zinc levels were seen in the IBS-D group compared to the controls (*p* = 0.001), which is associated with higher depressive symptoms and anxiety scores compared to healthy controls. A correlation between zinc status and psychological state in IBS patients has also been observed. Reduced absorption, enhanced zinc fecal excretion, and intestinal dysbiosis may explain the low levels of serum zinc in IBS patients. Gut dysbiosis may alter the functional capacity of the microbiota, resulting in reduced mineral absorption, short-chain fatty acid synthesis, and carbohydrate digestion. These alterations may decrease zinc bioavailability and trigger gastrointestinal and mental health disturbances. Moreover, serum zinc levels were significantly lower in the IBS-D group compared with the control group. Serum zinc was negatively correlated with serum zonulin in IBS-D patients, a marker of intestinal permeability (tight junction). Also, an inverse correlation between serum zinc levels and the prevalence of IBS-D was observed. A high prevalence of vitamin D deficiency was observed in patients with IBS. Serum 25(OH)D_3_ concentrations <20 ng/mL were reported in 66.7% of IBS patients. Moreover, vitamin D deficiency was linked with a higher severity of IBS symptoms and poor quality of life. Lower serum concentrations of 25(OH)D_3_ were correlated significantly with a higher severity of abdominal pain, intestinal gas, abdominal distension, overall digestive symptoms, and IBS-SSS [90,93,94].

### 5.5. Peppermint Oil for IBS Patients

Peppermint oil exerted clinical benefits in three RCTs, with significant improvement in digestive symptoms and improvement in IBS-SSS in patients, both children and adults, at a daily dose ranging from 250 to 540 mg/day [95,96,97].

## 6. Personalized Adjunct Care Program—Support for PRO Follow-Up of Digital Tool

Digital platforms enable the creation of personalized care programs, providing web content specific for IBS and IBD patients such as articles, videos on nutrition, diet, recipes, physical activity, and cognitive and behavioral therapies. Patient-Related Outcomes (PRO) are self-assessed by the patient on a series of scales for sleep quality, relationships with others, energy, anxiety, mood, work capacity, and joint pain, and may be followed across months with a platform for patients and one specific for healthcare practitioners. These PRO can be followed by healthcare practitioners. The development of digital platforms may be a good strategy for the set-up of personalized care programs for IBS and IBD patients and the follow-up of digestive symptoms and extra-digestive symptoms by the patients and healthcare practitioners for a coordinated patient care personalized program.

## 7. Conclusions

A Mediterranean diet, physical activity, and sessions of cognitive and behavioral therapies can be part of personalized care program for IBS and IBD patients, with demonstrated clinical benefits for digestive symptoms, quality of life, disease severity score, and risk of flare ups. Medical nutrition therapies including chitin glucan, selected probiotics, soluble fibers, and micronutrients may have added value for digestive symptom management and nutritional deficiency correction for these patients. The PRO self-assessment by the patients on digestive symptoms and quality of life components, as well as the follow-up by healthcare practitioners of such PRO, may be facilitated by the development of digital platforms with dedicated web content and specific access for patients and physicians.

Randomized controlled studies may also help assess the impact of the combination of Mediterranean diet and physical activity long term on digestive symptoms severity and frequency, as well as inflammatory markers and disease severity for IBS, UC, and CD. Also, randomized controlled trials on the combination of probiotics and soluble fibers for the future development of synbiotics as medical nutrition therapies that may be associated with micronutrients will be also helpful in this area of research, to build optimized care program for these patients. The establishment of minimal effective dose and the duration of use of such medical nutrition therapies depending on patient type and disease severity still need to be validated.

## Figures and Tables

**Table 1:** Efficacy data for several diets clinically tested for IBD patients and their strength of scientific evidence, (-): insufficient data, additional studies are required, +: low level of scientific evidence, ++: intermediate level of scientific evidence; +++: high level of scientific evidence.

Diets	Strength of Data	Clinical Data in IBS and IBD Patients
Fibers > 23.7 g/d vs. fibers < 10.4 g/d	++	A 40% reduction risk of a flare-up after six months in CD patients in comparison to those whose median fiber intake was 10.4 g/d.
Vegetarian Diet	+	CRP decrease. Higher remission rate than an omnivore diet.
Gluten-Free Diet	(-)	No conclusion can be drawn from the scientific data available.
SCD (Specific Carbohydrate Diet)	(-)	Limited number of RCTs or large observational studies. Potential digestive symptoms improvement—need additional studies to conclude.
Low FODMAP Diet	++	Several RCTs: improvement in stool frequency, and reduction in bloating, abdominal pain, and frequency of digestive symptoms (heartburn, bloating, intestinal gas, belching, nausea, and incomplete evacuation). However, it may be associated to nutritional deficiencies.
Mediterranean Diet	+++	Several clinical studies and meta-analyses: adherence score to the Mediterranean diet linked to decreased risk of UC and CD, significantly lower number of patients in active phase. Reduction in inflammatory markers. Beneficial modulation of intestinal flora, improvement in digestive symptoms.

**Table 2:** Summary of medical nutrition therapies for IBS and IBD patients in digestive symptoms management in addition to standard care.

Medical Nutrition Therapies	Clinical Findings of Randomized Controlled Trials (RCTs) on IBS or IBD Patients or International Clinical Practice Recommendations
Chitin glucan: 1.5–3 g/d 12 weeks	Reduced visceral pain perception, anti-inflammatory action at HED dose—involvement of MOR, CB2, and cytokine modulation (increased IL 10; reduced IL8, IL1b), sequestration of LPS bacterial toxins. Two RCTs on-going in 2024 on IBS and IBD patient with IBS-like symptoms. One RCT: microbiota modulation, transit normalization.
Selected probiotics *B. longum* 35624 *L. acidophilus* La 5 and NCFM *Lactobacillus Plantarum* CECT7484 *Lactobacillus Plantarum* CECT7485 *Bacillus coagulans*	Significant reduction in digestive symptoms (abdominal pain, bloating, bowel movement difficulty, etc.). Quality of life improvement. Reduced IBS severity score or composite score in IBS or IBD patients. Reduction in visceral hypersensitivity -> reduction of abdominal pain for some strains Reduction in serum inflammatory markers. Gut–brain axis modulation: serotonin reuptake/immunomodulation (nervous, immune, and endocrine communication pathways). Normalization of stool frequency and consistency. Dysbiosis reduction. USA, German French, Japan, British; Korean, Canadian, Romanian, Belgian, Indian recommendations in favor of probiotics use in clinical practice recommendations for IBS patients.
Micronutrients Vitamin D 50 µg/d Vitamin B6, B9, B12, B1, B2: 100% VNR Iron, Magnesium, Zinc: 30–100% VNR	Correction of nutritional deficiencies. Vitamin D, B6 and zinc status are linked to IBS severity score. Magnesium salts may improve stool consistency and frequency in patients. Zinc: intestinal immune modulation and intestinal gut barrier integrity. Vitamin B6, B9, and 12: intestinal immune modulation and cognitive health.
Soluble fibers: Human milk oligosaccharides, ispaghula, acacia gum, Fructo-oligosaccharides, Galacto-oligosaccharides (2–5 g/d)	Normalization of stool frequency and consistency. Significant improvement in digestive symptoms. Belgian and Indian clinical practice recommendations include soluble fibers for IBS patients.
Peppermint (*Mentha piperita*) oil (250–540 mg/d)	Three RCTs: Significant improvement in digestive symptoms + improvement in IBS Symptom severity scale score in IBS patients (children and adults)

## Data Availability

No new data were created (scoping review).
